# Validation of back‐calculated body lengths and timing of growth mark deposition in Hawaiian green sea turtles

**DOI:** 10.1002/ece3.2108

**Published:** 2016-04-06

**Authors:** Lisa R. Goshe, Melissa L. Snover, Aleta A. Hohn, George H. Balazs

**Affiliations:** ^1^ National Marine Fisheries ServiceSoutheast Fisheries Science CenterBeaufort Laboratory101 Pivers Island RoadBeaufortNorth Carolina28516; ^2^ National Marine Fisheries ServicePacific Islands Fisheries Science Center1845 Wasp BoulevardHonoluluHawaii96818

**Keywords:** *Chelonia mydas*, growth rate, line of arrested growth, mark–recapture, oxytetracycline, skeletochronology

## Abstract

Somatic growth rate data for wild sea turtles can provide insight into life‐stage durations, time to maturation, and total lifespan. When appropriately validated, the technique of skeletochronology allows prior growth rates of sea turtles to be calculated with considerably less time and labor than required by mark‐–recapture studies. We applied skeletochronology to 10 dead, stranded green turtles *Chelonia mydas* that had previously been measured, tagged, and injected with OTC (oxytetracycline) during mark–recapture studies in Hawaii for validating skeletochronological analysis. We tested the validity of back‐calculating carapace lengths (CLs) from diameters of LAGs (lines of arrested growth), which mark the outer boundaries of individual skeletal growth increments. This validation was achieved by comparing CLs estimated from measurements of the LAG proposed to have been deposited closest to the time of tagging to actual CLs measured at the time of tagging. Measureable OTC‐mark diameters in five turtles also allowed us to investigate the time of year when LAGs are deposited. We found no significant difference between CLs measured at tagging and those estimated through skeletochronology, which supports calculation of somatic growth rates by taking the difference between CLs estimated from successive LAG diameters in humerus bones for this species. Back‐calculated CLs associated with the OTC mark and growth mark deposited closest to tagging indicated that annual LAGs are deposited in the spring. The results of this validation study increase confidence in utilization of skeletochronology to rapidly obtain accurate age and growth data for green turtles.

## Introduction

Understanding growth rates, life‐stage durations, and age at maturation is critical for modeling sea turtle population trends and guiding management decisions for these threatened and endangered species (Heppell et al. 2003). However, knowledge of sea turtle length‐at‐age relationships, growth rates, and the factors influencing their variability is lacking (NRC 2010). Growth rate data are usually acquired through long‐term capture, mark, recapture studies of wild populations of sea turtles (Heppell et al. 2002). Mark–recapture studies typically require years of time and labor after initial tagging to yield growth‐rate calculations because sea turtles are highly migratory (reviewed by Musick and Limpus 1997), slow‐growing, and long‐lived (reviewed by Heppell et al. 2002). Analyzing growth marks retained in the bones of sea turtles through the technique of skeletochronology has provided an alternate and comparatively rapid means of acquiring a record of growth rates throughout life for individuals (Avens and Snover 2013).

Skeletochronology has been applied to several species of sea turtles, allowing estimates of life stages, growth rates, age at maturation, and total lifespan (reviewed by Avens and Snover 2013). Histological processing of cross‐sections of bones obtained from dead‐stranded turtles typically reveals growth cycles, with each represented as a wide, light zone of growth followed by a LAG (line of arrested growth), which is a period of little to no growth (reviewed by Avens and Snover 2013). The most recent growth occurs at the outside edge of the bone (Zug et al. 1986). Growth marks are retained in cortical bone and because the humerus has been shown to retain a high proportion of cortical bone, it is typically the focus of skeletochronology studies (Zug et al. 1986). Previous somatic rates throughout life can be back‐calculated from LAG dimensions when (1) the frequency of growth mark deposition has been validated; and (2) a proportional relationship exists between bone increment and somatic measurements (Chaloupka and Musick 1997). Individuals in the wild with tagging histories and injected with a bone marker, such as OTC (oxytetracycline), have provided validation of annual growth marks, as this allows calculation of deposition rate over a known time period (i.e., between bone marking and death). Such studies have validated annual growth marks in loggerhead sea turtles *Caretta caretta* in the western North Atlantic (Klinger and Musick 1992; Coles et al. 2001) and Hawaiian green turtles *Chelonia mydas* (Snover et al. 2011). Because the relationship between bone and somatic measurements may change throughout life, the complete size range of all ontogenetic stages must be represented when developing an equation that describes the relationship. Establishing such a relationship has allowed LAG diameters to be converted to previous carapace lengths for Kemp's ridley *Lepidochelys kempii* (Snover et al. 2007b), loggerhead (Snover et al. 2007a; Casale et al. 2011b; Piovano et al. 2011; Petitet et al. 2012; Avens et al. 2013, 2015), hawksbill *Eretmochelys imbricata* (Snover et al. 2013), olive ridley *Lepidochelys olivacea* (Zug et al. 2006; Petitet et al. 2015), and green turtles (Zug and Glor 1998; Goshe et al. 2010; Avens et al. 2012; Murakawa 2012). In tagged turtles, agreement between carapace lengths estimated or back‐calculated from the diameter of the LAG thought to be deposited closest to the time of tagging and those measured at the time of capture provides indirect validation of the frequency of LAG deposition because identification of the LAG associated with tagging is based on the assumption that one LAG is deposited each year (e.g., Avens et al. 2013). Close agreement between the two measures also verifies the reliability of back‐calculated carapace lengths. Taking the difference between successive carapace‐length estimates throughout time for each turtle yields annual somatic growth rates. Growth models can be applied to skeletochronologically derived growth‐rate data for length‐at‐age estimates, providing critical data for managing endangered and threatened sea turtle populations.

Skeletochronology relies on assumptions that must be validated for each sea turtle species, as well as populations inhabiting different regions. For green turtles in Hawaii, annual LAG deposition has been confirmed (Snover et al. 2011) and a proportional relationship between bone and carapace measurements has been described for size classes that included juveniles and adults (Murakawa 2012). However, the accuracy of back‐calculated carapace lengths has yet to be verified for this population. The goal of the present study is to validate skeletochronological estimates of body length for Hawaiian green turtles with a prior history of tagging, OTC injection, and measurement. Agreement between the estimated and actual carapace lengths measured at tagging would validate the ability to calculate prior growth rates quickly and accurately using the growth marks retained in bones. Additionally, we compare OTC mark and LAG placement with the goal of identifying the time of year of LAG deposition for green turtles in Hawaii.

## Methods

Humeri were recovered from 10 Hawaiian green turtles from the wild that had previously been measured, tagged, and injected with the bone marker OTC during mark–recapture studies between 1994 and 2000 (Balazs 1999; Balazs and Chaloupka 2004) or prior to release after stranding alive. Turtles had either dead‐stranded or stranded alive and were euthanized by a veterinarian if recovery was not deemed possible (see Snover et al. 2011 for stranding condition and histories for turtles from which samples were recovered). Observers measured standard SCL (straightline carapace length; Wyneken 2001) both at capture (or live stranding) and after dead recovery. Inconel and passive integrated transponder tags applied at the first encounter allowed individual turtles to be identified. The presence or absence of fibropapilloma tumors, which occur externally on green turtles (Herbst 1994), and the severity of the disease was recorded both at capture and recovery (Snover et al. 2011).

### Back‐calculation of carapace lengths

Samples in the current study were histologically processed for a previous skeletochronological study (Snover et al. 2011). Following the methods of Snover and Hohn (2004), humeri were prepared for skeletochronological analysis of growth marks. Digital images of decalcified, stained cross‐sections were taken sequentially at 4x magnification using an Olympus BX41 trinocular compound microscope (Olympus America Inc., Melville, New York, USA), Olympus Colorcube‐12 Color CCD digital camera, and Olympus Microsuite Image Analysis software. These digital images were then manually stitched together using Adobe Photoshop for a composite image. MLS, AAH, and when no consensus was reached, LRG, identified LAGs by tracing them around the circumference of each cross‐section using the composite digital images.

Because growth marks are deposited annually in Hawaiian green turtles (Snover et al. 2011), we assigned calendar years to each LAG, with the most recent year assigned to the LAG closest to the outer circumference of the bone. The LAG deposited closest to the timing of tagging was identified and LAG diameter was measured along an axis parallel to the dorsal edge of the cross‐section using the composite digital images. SCL at tagging was estimated using the back‐calculation equation from Murakawa (2012) for 167 Hawaiian green turtles ranging from 36.4 to 97.9 cm SCL, which incorporated the body proportional hypothesis (Francis 1990):

(1) $$L=\left[L_{\mathrm{op}}+b{\left(D-D_{\mathrm{op}}\right)}^{c}\right]\left[L_{\mathrm{final}}\right]{\left[L_{\mathrm{op}}+b{\left(D_{\mathrm{final}}-D_{\mathrm{op}}\right)}^{c}\right]}^{-1}$$

The estimated SCL is *L*,* L*_op_ is the average hatchling SCL, *D* is the LAG diameter of interest, *D*_op_ is the minimum hatchling humerus diameter, *L*_final_ is the SCL measured at dead recovery, *D*_final_ is the diameter of the humerus cross‐section, *b* is the slope, and *c* is the proportionality coefficient. Values from Murakawa (2012, S. K. K. Murakawa National Marine Fisheries Service, personal communication) were used for average hatchling SCL (5.1 cm), hatchling humerus diameter (2.6 mm), slope (*b *=* *3.127), and proportionality coefficient (*c *=* *0.928).

Only those turtles with measureable LAG diameters deposited closest to the time of tagging and measurements taken in the field at tagging were included. The estimated and actual SCLs at tagging were compared using a paired‐sample Wilcoxon rank sum test (Zar 2010). Turtles with and without visible OTC marks were included because the visibility of an OTC mark in the bone was irrelevant to this analysis.

Straightline carapace lengths were back‐calculated using the OTC mark diameters (*as described below*) for those turtles with measurable OTC marks. These back‐calculated SCLs were compared to those measured by observers at the time of OTC injection and the difference and standard error were calculated.

### Timing of growth‐mark deposition

Establishment of a back‐calculation equation (Murakawa 2012), validation that LAGs are annual (Snover et al. 2011), ability to back‐calculate SCLs from growth mark diameters (see Back‐calculation of carapace lengths *section, above*), and OTC injection made it possible to examine the timing of LAG deposition in Hawaiian green turtles. OTC injection is typically recorded in the bones as a fluorescent line visible in cross‐sections viewed under ultraviolet light (Milch et al. 1958). Because turtles were previously injected with OTC, it was possible to associate a fluorescent mark in their bones with a known date. The time of year that LAGs were deposited could then be inferred by examining where the yearly LAGs were deposited in the bone relative to the OTC mark.

To determine the placement of LAGs associated with specific years relative to the OTC mark, we measured LAG diameters and back‐calculated SCLs as previously described. Unprocessed humerus cross‐sections from each turtle were examined for OTC marks, as described by Snover et al. (2011). Like the stained sections, digital images were stitched together for composite images of the unprocessed bones viewed under ultraviolet light. OTC mark diameters were measured on the calibrated digital composite images using Olympus Microsuite (Olympus America Inc.) and corresponding SCLs were back‐calculated using eqn (1). The back‐calculated SCLs of both the OTC marks and LAG diameters representing the years closest to OTC injection dates were then compared to determine if LAGs had been deposited before, after, or at approximately the same time as OTC injection.

In a previous study (Snover et al. 2011), placement of these marks was determined by finding landmarks along the perimeters of both the stained and OTC sections to identify matching regions, photographing those regions, and measuring the distance from the outer margin of the bone to the OTC mark. This distance was then noted on the image of the stained section to identify where the OTC mark was deposited in relation to the LAGs. Our current methods differed from Snover et al. (2011) in that we acquired and compared LAG and OTC diameter measurements, which allowed for a greater level of accuracy and precision in determining the exact placement of the marks relative to each other.

## Results

### Back‐calculation of carapace lengths

Ten turtles had been measured at the time of tagging and had measureable LAG diameters deposited close to the time of tagging. Prior to recovery, turtles spent between 0.58 and 9.35 years at large. The sample included turtles with (*n* = 6) and without (*n* = 4) fibropapilloma tumors (see Snover et al. 2011).

At the time of tagging, their SCL measurements ranged from 44.7 to 83.8 cm (mean ± SD = 58.8 ± 12.3). Their back‐calculated SCLs at tagging ranged from 44.3 to 84.6 cm (mean ± SD = 59.0 ± 12.3). There was no significant difference between SCLs measured at tagging and estimated SCLs back‐calculated from LAGs deposited closest to the date of tagging (Table 1, Wilcoxon rank sum test, *T *=* *8.5, *N *=* *7, *P *>* *0.20). This validation allows prior growth rates to be calculated from successive LAG diameters of Hawaiian green turtles. The close agreement between back‐calculated SCLs and those measured at tagging also provides indirect validation that LAGs are deposited annually in green turtles between 44.7 and 85.5 cm SCL.

**Table 1:** Comparison of carapace lengths measured at tagging with those back‐calculated from growth mark diameters within stained humerus cross‐sections from Hawaiian green turtles *Chelonia mydas*. Date is day‐month‐year. SCL = straightline carapace length (cm). LAG = line of arrested growth.

ID	Date stranded	SCL at stranding	Date tagged	SCL at tagging	Estimated SCL at tagging	Year LAG deposited	Difference in SCL
CM‐1	8‐2‐99	58.5^a^	23‐4‐97	55.1	55.1	1997	0.0
CM‐4^e^	10‐12‐99	69.6	21‐12‐98	69.4	69.6	1999	−0.2
CM‐6	9‐5‐00	56.3	5‐10‐99	55.9	56.3	2000	−0.4
CM‐8	20‐2‐01	70^b^	24‐4‐00	70.6	70.0	2000	0.6
CM‐9^e^	23‐4‐01	44.5^b^	14‐4‐00	44.7	44.3	2000	0.4
CM‐10^e^	11‐12‐01	85.5	25‐4‐00	83.8	84.6	2000	−0.8
CM‐11^e^	28‐3‐02	57.2	8‐7‐97	54.8	54.8	1997	0.0
CM‐12^e^	17‐10‐02	60.2	12‐5‐98	57.4	57.7	1998	−0.3
CM‐13	19‐10‐03	69.5	17‐6‐94	45.5	47.0^c^	1994	−1.5
CM‐14^e^	14‐7‐04	53.7	16‐5‐00	51	51.0	2000	0.0
					Mean absolute difference:		0.4 (0.3)^d^

a SCL at stranding was not measured. SCL is that measured when turtle was captured 3 months before death.

b Possible observer measurement error (SCL) or shrinking turtle; SCL measured at stranding was less than that measured at tagging.

c SCL back‐calculated using the oxytetracycline mark diameter; 1994 LAG within resorption core and not measurable in stained cross‐section.

d Mean absolute difference calculated without CM‐13.

e Fibropapilloma tumors were present.

Straightline carapace lengths were back‐calculated for the five turtles (51–69.4 cm SCL) that retained measurable OTC mark diameters and were compared to the SCLs measured by observers at the time of OTC injection. We found a mean absolute difference of 0.3 cm SCL between measures with a standard error of 0.1 cm SCL (Table 2).

**Table 2:** Comparison of back‐calculated straightline carapace lengths (SCLs) of OTC (oxytetracycline) marks and corresponding LAG (line of arrested growth) diameters representing the years closest to OTC injection dates to assess the timing of LAG deposition in Hawaiian green sea turtle *Chelonia mydas* humeri. Date is day‐month‐year. Parentheses indicate the difference and direction of error between SCLs measured at OTC injection and back‐calculated from the diameter of the OTC mark with the mean absolute difference and SE (standard error)

ID	OTC date	OTC section back‐calculated SCL	Stained section back‐calculated SCL	LAG year
CM‐1	23‐4‐97	55.9 (−0.8)	55.1	1997
CM‐4	21‐12‐98	69.4 (0.0)	69.6	1999
CM‐11	8‐7‐97	55.0 (−0.2)	54.8	1997
CM‐12	12‐5‐98	57.7 (−0.3)	57.7	1998
CM‐14	16‐5‐00	51.0 (0.0)	51.0	2000
Mean difference ± SE		0.3 ± 0.1		

### Timing of growth‐mark deposition

**Figure 1:**
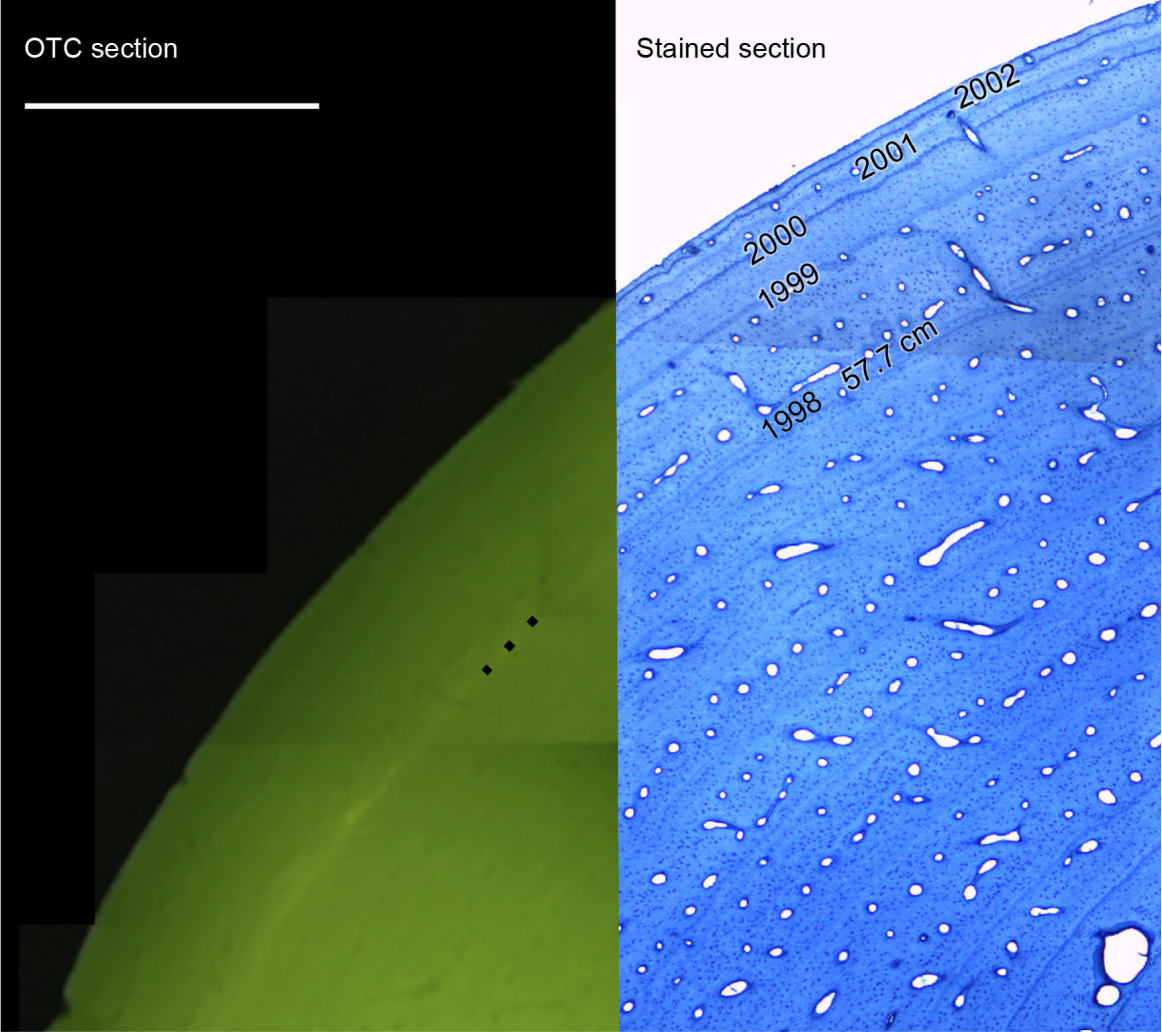
Green turtle humerus cross‐section for CM‐12 viewed under ultraviolet light with fluorescent OTC (oxytetracycline) mark visible (indicated by black dotted line) next to stained cross‐section with years assigned to growth marks. Turtle was OTC injected on 12 May 1998 and measured 57.4 cm SCL (straightline carapace length). 1998 LAG (line of arrested growth) was a closely spaced double interpreted as 1 annual LAG according to Goshe et al. (2010) and Snover et al. (2011). Both the diameter of the OTC mark and 1998 LAG yielded a back‐calculated SCL of 57.7 cm, indicating that the timing of deposition for each was similar. White bar indicates 1 mm.

Both the diameters of the OTC mark and the LAG deposited closest to tagging were measureable for five turtles ranging from 51 to 69.4 cm SCL at the time of tagging (mean ± SD = 57.5 ± 7.0). These turtles were injected with OTC between the months of April and December. The back‐calculated SCLs indicated that annual LAGs were deposited after December (CM‐4) and before mid‐May, as the diameters of the OTC mark and annual LAG were equivalent for both of the turtles injected with OTC in May (CM‐12, CM‐14; Fig. 1, Table 2). However, the exact timing of LAG deposition varied by individual, sometimes occurring prior to May (CM‐1). These results support the assumption that LAG deposition occurs in the spring for Hawaiian green turtles.

## Discussion

Our results indicate that prior carapace lengths can be reliably back‐calculated from growth marks retained in the humeri of Hawaiian green turtles. The mean difference of 0.4 cm SCL between carapace lengths measured at tagging and those back‐calculated using LAG diameters was less than that reported in similar studies (Snover et al. 2007a; Goshe et al. 2010; Avens et al. 2012, 2013, 2015). As Snover et al. (2007a) speculated, the difference might be explained by observer measurement error as well as a difference in the timing of when LAGs were deposited and carapace lengths were measured. OTC marking offered a rare opportunity to examine the difference between back‐calculated and measured SCLs, because the OTC mark was deposited at the same time as observers collected a SCL measurement. The mean difference between these measures was low, at 0.3 cm SCL, for the turtles with measureable OTC mark diameters. Nonetheless, these small differences will not appreciably affect rapid and accurate calculation of growth rates through the conversion of successive LAG diameters to prior carapace lengths using skeletochronology.

In comparing carapace lengths at live capture and dead recovery, two turtles appeared to have decreased in length (CM‐8 and CM‐9). Both were noted as emaciated at stranding. Negative growth rates have been documented in sea turtles (Braun‐McNeill et al. 2008; Bjorndal et al. 2013) and may be the result of observer measurement error of SCL, damage to the carapace, or a decrease in carapace length between the two events. Shrinking body length has also been observed in marine iguanas *Amblyrhynchus cristatus* under low‐food conditions (Wikelski and Thom 2000) and in tortoises (Field et al. 2007; Loehr et al. 2007).

Annual LAG deposition was directly verified in Hawaiian green turtles ranging from 45.5 to 69.6 cm SCL in a previous study (Snover et al. 2011). Van Houtan et al. (2014) identified the need for validation of annual marks outside of this size range. The current study extends the carapace length in which annual LAGs have been verified to 85.5 cm SCL, as we were able to indirectly validate annual LAG deposition in CM‐10, which was not included in Snover et al.'s (2011) direct validation due to the lack of a visible OTC mark. Under the assumption of one mark per year, close agreement of back‐calculated and measured carapace lengths at tagging offered indirect validation of annual growth marks in CM‐10, which measured 83.8 cm SCL at tagging and 85.5 cm SCL at stranding. Through indirect validation, we demonstrated not only that annual LAGs were present in humeri but also that the carapace lengths estimated using LAG diameters were comparable to those measured during mark–recapture studies. In the Hawaiian green turtle population, length at maturation ranges from 75 to 106 cm SCL (Balazs et al. 2015). Therefore, skeletochronology can now be used to provide back‐calculated annual growth rates for Hawaiian green turtles that are within the size range of mature individuals.

Double LAGs were identified in CM‐14 and interpreted by Snover et al. (2011) as annual. These LAGs typically appeared as a dark, continuous line with a closely spaced lighter line when tracked around the circumference of the section. Our results support Snover et al.'s (2011) interpretation of double LAGs as an annual mark, as we found no difference between the SCL measured at tagging in May 2000 and the back‐calculated carapace length from the LAG assumed to have been deposited in 2000 for CM‐14. Other turtles in this sample also had double LAGs that were interpreted as representing a single year. Our SCL back‐calculation results support this interpretation in those turtles and suggest that double LAGs may be common in Hawaiian green turtles. Double LAGs deposited in a single year have been described in other vertebrates (Klevezal 1996) and although the present study cannot explain why these marks are deposited, our results indicate that this is the correct interpretation of such marks in Hawaiian green turtles.

Line of arrested growths were deposited in turtles with and without varying degrees of fibropapilloma. Because severe fibropapilloma is known to result in lower growth rates in green turtles (Chaloupka and Balazs 2005), this slowed growth would be expected to result in LAGs that are spaced closer together. Our results suggest that LAG deposition is not otherwise affected by fibropapilloma, as SCL was reliably back‐calculated for these turtles (Table 1).

Understanding of the time of year that LAGs are deposited is important in the application of skeletochronology, as this allows calendar years to be assigned more accurately to individual LAGs based on the time of year a turtle stranded dead. For example, if LAG deposition is known to occur in the spring, the calendar year assigned to the final LAG deposited in spring‐stranded turtles would be the same as the year of stranding if there is little to no differentiation between the final LAG and outer edge of the bone; the previous calendar year would be assigned if the amount of growth between the final LAG and outer edge is >0 (see supplement of Avens et al. 2013). In the present study, we found that two turtles that had been OTC injected in May (CM‐12 and CM‐14) deposited a LAG at approximately the same time as the OTC mark, while the results from Snover et al. (2011) indicated that the same LAGs were deposited slightly before the OTC marks. The technique we used in the present study to determine the placement of the OTC mark relative to LAGs was different in that we were able to measure and compare total diameters of both the OTC mark and LAG of interest, which should offer more accurate results than the method previously used (Snover et al. 2011). This difference did not affect the conclusion of either study that one LAG per year was deposited after OTC marking.

This is the first study to confirm the assumption that LAG deposition occurs in the spring for green turtles. The five turtles with measureable OTC mark diameters and corresponding LAGs indicated that like Kemp's ridley and hawksbill sea turtles in the Northern Hemisphere, LAG deposition also occurs in the spring for this species (Snover and Hohn 2004; Snover et al. 2013). Although we were not able to determine the exact timing, our results suggest that annual LAG deposition occurs after December and before mid‐May, with individual variability. The timing of LAG deposition is likely influenced by endogenous cycles driven by environmental factors (Castanet et al. 1993). While evidence suggests that seasonal changes in photoperiod influence LAG deposition in primates (Castanet et al. 2004), similar studies on reptiles are needed.

## Conclusions

To understand sea turtle population trends, data are required on basic biological parameters such as age at maturation and growth rates throughout life (NRC 2010). Skeletochronology is one of several methods available to accurately estimate these parameters. Estimates of sea turtle age at maturation using skeletochronology demonstrated the utility of this approach in the Mediterranean, as they converged with estimates yielded by mark–recapture studies and length‐frequency analysis (Casale et al. 2009, 2011a,b). Avens et al. (2015) also demonstrated that sea turtle growth rates estimated using skeletochronology converged with those from mark–recapture studies in the western North Atlantic. The results of the present study indicate that skeletochronology produces reliable estimates of prior carapace lengths and provides additional validation for annual LAG deposition, which allows future studies to use this technique with confidence to fill gaps in the current knowledge of growth rates of Hawaiian green turtles.

## Conflict of Interest

None declared.
